# Association of soluble urokinase plasminogen activator receptor levels with fibrotic and vascular manifestations in systemic sclerosis

**DOI:** 10.1371/journal.pone.0247256

**Published:** 2021-02-22

**Authors:** Sheraz Butt, Jørgen L. Jeppesen, Line Vinderslev Iversen, Mogens Fenger, Jesper Eugen-Olsen, Charlotte Andersson, Søren Jacobsen

**Affiliations:** 1 Department of Internal Medicine, Amager and Hvidovre University Hospital, Glostrup, Denmark; 2 Department of Dermatology, Bispebjerg University Hospital, Copenhagen, Denmark; 3 Department of Dermatology and Allergy, Odense University Hospital, Odense, Denmark; 4 Department of Clinical Biochemistry, Amager and Hvidovre University Hospital, Hvidovre, Denmark; 5 Clinical Research Centre, Amager and Hvidovre University Hospital, Hvidovre, Denmark; 6 Department of Cardiology, Herlev-Gentofte University Hospital, Hellerup, Denmark; 7 Copenhagen Lupus and Vasculitis Clinic, Centre for Rheumatology and Spine Disease, Rigshospitalet, Copenhagen University Hospital, Copenhagen, Denmark; Medical University of South Carolina, UNITED STATES

## Abstract

**Objective:**

We assessed the association of suPAR (soluble urokinase plasminogen activator receptor) plasma levels with fibrotic and vascular manifestations in patients with systemic sclerosis (SSc).

**Methods:**

suPAR plasma levels were measured in 121 consecutive patients with SSc and correlated to pulmonary and vascular features of SSc, including interstitial lung disease as characterized by percentage of predicted CO diffusing capacity (DLco) and forced vital capacity (FVC), pulmonary fibrosis by computed tomography, and pulmonary arterial hypertension, telangiectasias, and digital ulcers.

**Results:**

Overall, 121 SSc patients (84% females; mean age, 57 ± 12 [range: 22–79] years) were enrolled; 35% had diffuse cutaneous SSc. suPAR plasma levels ranged from 1.3–10.2 [median: 2.9 (p25–p75: 2.3–3.9)] ng/mL. Log(suPAR) levels correlated with DLco (r = -0.41, *p* <0.0001) and FVC (r = -0.26, *p* = 0.004), also when adjusted for age, sex, and pulmonary hypertension. A suPAR cut-off level of >2.5 ng/mL showed a sensitivity of 91% for identifying patients with either DLco <50% or FVC < 60% of the predicted values. Similarly, 19 (90%) had a suPAR >2.5 ng/mL among those diagnosed with pulmonary fibrosis vs. 59 (60%) among those who did not (*p* = 0.008). suPAR values were not associated with vascular manifestations.

**Conclusion:**

suPAR levels strongly correlated with pulmonary involvement in SSc. Future studies should test if suPAR estimation can be used for surveillance of severe pulmonary involvement in SSc.

## Introduction

Systemic sclerosis (SSc, scleroderma) is a rare autoimmune disease affecting nearly all organ-systems with substantially increased morbidity and mortality [1, 2]. Microvascular impairment, progressive fibrosis, and innate and adaptive immune dysregulation are hallmark pathophysiological features of SSc. The disease presents as several highly variable phenotypes [3, 4]. Therefore, there is an unmet demand for biomarkers that can reflect disease activity and severity in affected patients with severe fibrotic and vascular complications (such as interstitial lung disease and pulmonary hypertension). In this context, a number of hematological biomarkers indicative of interstitial lung disease, including surfactant protein, monocyte chemoattractant protein-1, interleukin-34, CCL-18, CXCL, and Kerbs von Lungren (KL)-6, have been identified [5–7]. Nevertheless, the clinical role of these biomarkers in the assessment of fibrotic manifestations (e.g., interstitial lung disease) or vasculopathy in SSc remains unclear. Furthermore, these tests are generally unavailable for routine clinical use.

C-reactive protein (CRP) is used as a general inflammatory marker in patients with autoimmune diseases. Prior studies have suggested that the pro-inflammatory interleukin-6 exerts transcriptional control on CRP release, and consequently, its blood levels. An elevated CRP level may therefore be associated with certain inflammatory SSc phenotypes, e.g. those involving progressive skin and lung involvement [8–12]. However, the diagnostic and prognostic sensitivity and specificity of a non-specific acute phase reactant, such as CRP may be limited, as it is not directly involved in SSc pathophysiology. Conversely, mechanistic molecular biomarkers that reflect biological dysregulation in pathogenic pathways specific to the disease promise to have more predictive potential for clinical and therapeutic management [3].

The urokinase plasminogen activator receptor (uPAR) is considered an important factor in the regulation of proteolysis, degradation of extracellular matrix, angiogenesis, and inflammation. An experimental deficiency of uPAR has been demonstrated to decrease angiogenesis and alter endothelial cell morphology [13]. Abnormal protein cleavage resulting in dysfunctional uPAR-pathways affects several immune mechanisms adversely, e.g. for differentiation of fibroblasts into myofibroblasts, an important step causing excessive production and deposition of extracellular matrix in SSc [14]. Systemic levels of soluble uPAR (suPAR) have been shown to correlate positively with immune system activation, with observational studies showing a positive correlation of this protein with both prognosis and mortality in a wide range of diseases [15]. When compared to many other inflammatory biomarkers, suPAR is biologically and chemically more stable both in vivo [16] and in vitro [17] and remains unaffected by the circadian rhythm or analytical preparations [17, 18]. A recent study demonstrated a possible association of suPAR levels with interstitial pulmonary and vascular involvement (e.g. development of pulmonary hypertension) in SSc patients as compared to those of healthy controls [19]. These data suggest that despite the fact that there is a possibility of an association between suPAR and systemic sclerosis, few studies have assessed this association. Therefore, in the present study, we measured suPAR levels and determined their clinical correlations with fibrotic and vascular complications occurring in patients with SSc.

## Methods

This cross-sectional and analytical study included 121 consecutive SSc patients fulfilling the American College of Rheumatology criteria for classification of the disease [20]. All data were collected in 2009 (January until February) to investigate the correlation of selected biomarkers with subclinical organ damage, as detailed in previously published studies [21, 22]. The patients were recruited at the outpatient clinics at the Department of Dermatology, Bispebjerg Hospital, and at the Department of Rheumatology, Rigshospitalet, both part of Copenhagen University Hospitals. All but one patient was of Western European descendants. Clinical examinations were performed at the respective departments. The biochemical analysis of suPAR were performed at Amager and Hvidovre University Hospital, Copenhagen, Denmark. The patients were informed on the project by mail or during the planed clinical consultation. Patients were subscribed only after having fully understood the benefits and risks of the project and after having been given time for consideration.

Patients who had not received biological medications within the last 6 months and were free of cancer, pregnancy, or systemic infections at the time of screening were included in the study. The population of SSc patients is considered representative of a larger cohort of patients with this disease. Demographical data found in this population is in accordance with demographical data from other SSc populations [23].

The study was approved by the local ethics committee (“De Videnskabsetiske Komiteer for Region Hovedstaden”, approval number H-B-2008-131) and carried out in accordance with the principles of the Declaration of Helsinki. All patients provided written, informed consent for study participation.

### Patients

All patients were screened and investigated by a single investigator, thereby eliminating the possibility of inter-individual variations in observations and evaluations. All patients were screened for the presence of abnormal skin thickness in 17 integumentary regions at the time of enrolment, and the results of this assessment were compiled and expressed in the form of the modified Rodnan skin score [24]. The relevant medical history of all patients was elicited by screening their medical records and by interviewing and recording reported comorbidities. The duration of disease was calculated as the time period from the onset of first non-Raynaud’s manifestation. Classification of SSc as diffuse or limited was performed in accordance with the definitions provided by LeRoy et al. [25].

Pulmonary function tests had been performed as a part of the usual clinical evaluation and the recorded results of all patients were ≤1-year-old at baseline. Data of forced vital capacity (FVC) and diffusing lung capacity of carbon monoxide (DLco) were also collected for the present study and are expressed as percentages (%) of predicted values. Diffusion capacity was estimated using a single-breath carbon monoxide and helium-carbon monoxide dilution principle and was standardized according to the patient’s hemoglobin concentration at the time. Blood pressures were measured at rest using standard electronic blood pressure machines. Hypertension was defined as a blood pressure >140/90 mm Hg.

### Procedures

We measured suPAR and high sensitivity CRP (hsCRP) levels in thawed plasma that had been collected in 2009. Whole blood (drawn from venipuncture of the cubital fossa) was centrifuged immediately after extraction and subsequently plasma samples were stored at −80°C until the analysis of suPAR levels. Levels of suPAR were measured using the commercially available suPARnostic^®^ kit (ViroGates, Copenhagen, Denmark). Intra- and inter-assay variations in these estimations were reportedly low (<10%). The suPAR levels have been shown to be stable in spite of freezing and thawing of plasma samples [26]. The hsCRP levels were estimated using a standard clinical platform, as in previous studies [27].

### Statistical analysis

Baseline characteristics are expressed as means and standard deviations (continuous variables) or as percentages (discrete variables). Due to the skewed distribution of concentrations of suPAR and hsCRP, the estimated variables were log-transformed (base 10) before analysis. Associations between suPAR and continuous variables were determined by means of Pearsons correlation analysis. Given a type 1 error rate of 0.05, a type 2 error rate of 0.20 and the study sample size, allowed us to be conclusive on correlation coefficients down to +/- 0.25. Linear regression analysis was used to analyze sex- and age-adjusted associations. To evaluate the existence and level of associations of suPAR and hsCRP concentrations with those of other variables, generalized linear models were applied. Two-sided *p*-values <0.05 were considered statistically significant. All analyses were performed using SAS studio^®^ or the SAS version 9.4 (Cary, North Carolina, USA) software.

## Results

### Baseline characteristics

**Fig 1:**
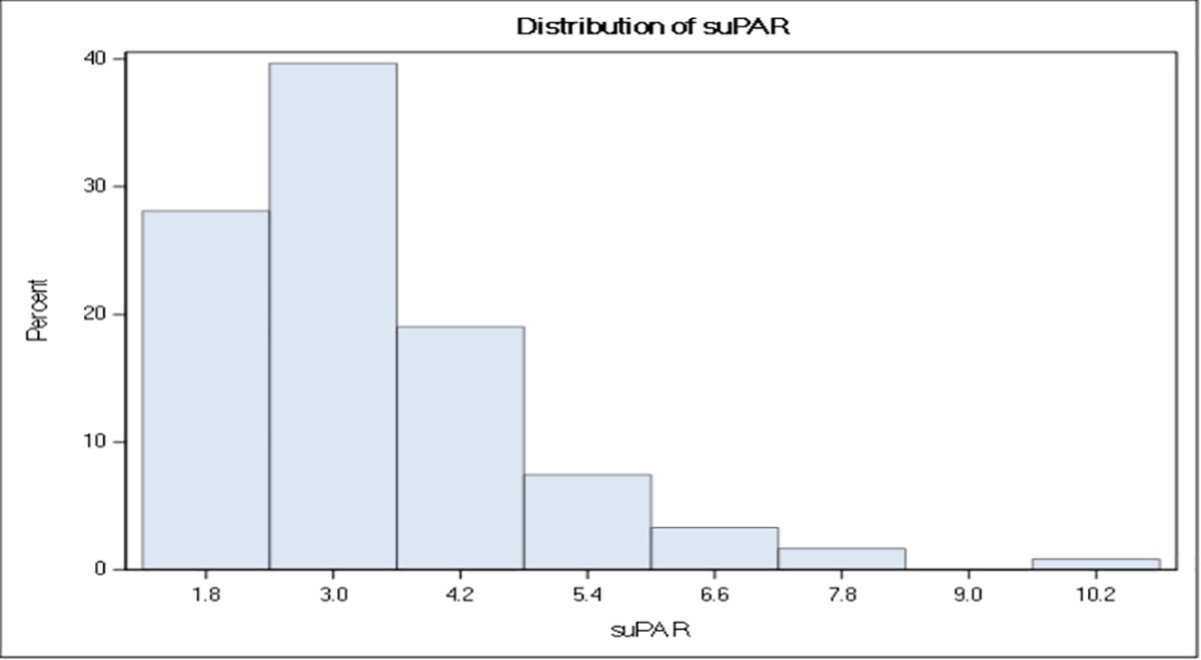
Concentration range of suPAR. Histogram showing the distribution of suPAR concentrations (ng/mL) in the study population.

A total of 121 patients (including 120 white) were enrolled. Of these, 84% were females. The mean age with standard deviation (± SD) of the study group was 57 ± 12 (range: 22–79) years. The average disease duration of the group was 12 ± 9 (range: 0–53) years, and 35% and 65% of the study patients had diffuse cutaneous and limited cutaneous SSc, respectively. The concentrations range of suPAR was 1.3–10.2 ng/ml (median: 2.9, p25–p75: 2.3–3.9 ng/ml) and demonstrated a skewed distribution (**Fig 1**).

Baseline characteristics are presented in **Table 1**. Log suPAR was significantly associated with age, smoking and hsCRP (log10base). Furthermore, log suPAR was significantly associated with fibrotic features of SSc (modified Rodnan skin score, diffuse cutaneous SSc, radiographically confirmed lung fibrosis, FVC, DLco).

**Table 1:** Baseline characteristics of 121 patients with Systemic Sclerosis (SSc).

Characteristics	N	r (*p*)	beta (*p*)
Sex, female (%)	102 (84%)	-0.08 (0.37)	-0.08 (0.37)
Age, years, mean (SD)	57 (12)	0.22 (0.01)	6.39 (0.01)
Disease duration, years, mean (SD)	11.7 (8.9)	0.00 (0.99)	-1.09 (0.59)
Body mass index, mean (SD)	23.9 (3.8)	-0.06 (0.51)	-0.50 (0.57)
Current smoker, n (%)	27 (25%)	0.33 (<0.001)	0.37 (<0.001)
hsCRP, mg/l, mean (SD)	1.26 (1.21)	0.33 (<0.001)	1.10 (<0.001)
Anti-SCL70-antibodies, n (%)	15 (13%)	0.06 (0.54)	0.05 (0.48)
Centromere-antibodies, n (%)	50 (42%)	0.11 (0.23)	0.06 (0.56)
RNA-polymerase-III-antibodies, n (%)	13 (11%)	0.00 (0.98)	0.05 (0.49)
Modified Rodnan Skin, Score^20^, n (%),	11 (9)	0.12 (0.20)	4.29 (0.02)
Type of SSc, diffuse cutaneous, n (%)	42 (35%)	0.12 (0.18)	0.22 (0.04)
Past or present cutaneous ulcers, n (%)	45 (38%)	0.00 (0.96)	-0.01 (0.95)
FVC, % of predicted value, mean (SD)	96.7 (21.1)	-0.27 (0.004)	-15.67 (0.001)
DLco, % of predicted value, mean (SD)	64.6 (19.4)	-0.41 (<0.0001)	-18.75 (<0.0001)
Radiographically confirmed lung fibrosis, n (%)	21 (18%)	0.25 (0.007)	0.23 (0.01)
Evidence of pulmonal hypertension on echocardiography, n (%)	19 (17%)	0.16 (0.09)	0.13 (0.14)
Telangiectasia, n (%)	40 (34%)	-0.16 (0.08)	-0.09 (0.43)
Hypertension, n (%)	30 (25%)	-0.06 (0.48)	-0.02 (0.82)
Systolic blood pressure, mm Hg, mean (SD)	138 (23)	-0.05 (0.62)	-4.74 (0.36)
Diastolic blood pressure, mm Hg, mean (SD)	77 (12)	-0.10 (0.28)	-3.01 (0.28)
History of thrombosis, n (%)	19 (17%)	-0.22 (0.02)	-0.18 (0.05)
History of coronary artery disease, n (%)	8 (7%)	-0.04 (0.74)	-0,02 (0.79)
History of intermittent claudication, n (%)	3 (2.5)	-0.07 (0.50)	-0.02 (0.68)

Demographic and clinical characteristics as well as association to logarithm of suPAR plasma levels by unadjusted and adjusted (sex and age) analyses.

Percentages are calculated based on the number of individuals without missing values. The maximal number of individuals with missing values was 32 (for medical history of coronary artery disease). For the other (non-medical history) variables, most patients had complete data: three individuals had missing values of pulmonary function tests, six individuals had missing data for pulmonary hypertension on echocardiography, and seven individuals had missing data on RNA-polymerase-III-antibodies. (SSc: systemic sclerosis; suPAR: soluble urokinase plasminogen activator receptor; hsCRP: high sensitivity C-reactive protein; FVC: forced vital capacity; DLco: lung diffusing capacity of carbon monoxide; mRSS: modified Rodnan skin score).

### Associations with vascular involvement

Vascular involvement was not associated with suPAR levels. There were no significant differences in logarithmic suPAR levels between individuals presenting with and without pulmonary hypertension (mean: 1.26 vs. 1.1, *p* = 0.09), telangiectasias (mean: 1.1 vs. 1.0, *p* = 0.08), or past or present digital ulcers (mean: 1.1 vs. 1.1, *p* = 0.96). Furthermore, the logarithmic hsCRP levels were also found to be comparable between patients with and without pulmonary hypertension (*p* = 0.60), telangiectasias (*p* = 0.40) and digital ulcers (*p* = 0.39).

### Exploratory analyses

To determine whether suPAR could be used as a screening biomarker for more severe pulmonary involvement in SSc, we estimated the concentrations of suPAR in patients with and without either diffusion capacity of <50% or forced vital capacity of <60% of predicted values. In total, 32 (27%) patients were found to have severe pulmonary involvement according to this definition. Median (p25–p75) concentrations of suPAR were estimated at 3.8 (3.0–4.6) ng/mL and 2.6 (2.1–3.4) ng/mL for patients with and without severe pulmonary involvement, respectively. A cut-off value >2.5 ng/mL encompassed 29 of the 32 patients, corresponding to a sensitivity of 91% and a negative predictive value of 96%. In total, 41 (34%) patients included in the study had suPAR values ≤2.5 ng/mL. Of those without severe pulmonary involvement (n = 80, 73%), 38 were correctly classified as not having severe pulmonary disease according to their suPAR concentrations, corresponding to a specificity of 43%. Similarly, 19 of 21 (90%) patients diagnosed with pulmonary fibrosis on X-ray and high-resolution computed tomography (CT), had suPAR values >2.5 ng/mL as compared to 59 of 98 (60%) of those who did not show any radiological signs of the condition (*p* for difference = 0.008). Further, among those with fibrosis on imaging (X-ray), of which only 7 underwent high-resolution computed tomography, unadjusted and adjusted linear regression models showed no significant association between suPAR and FVC/DLco.

### Associations with pulmonary involvement

**Fig 2:**
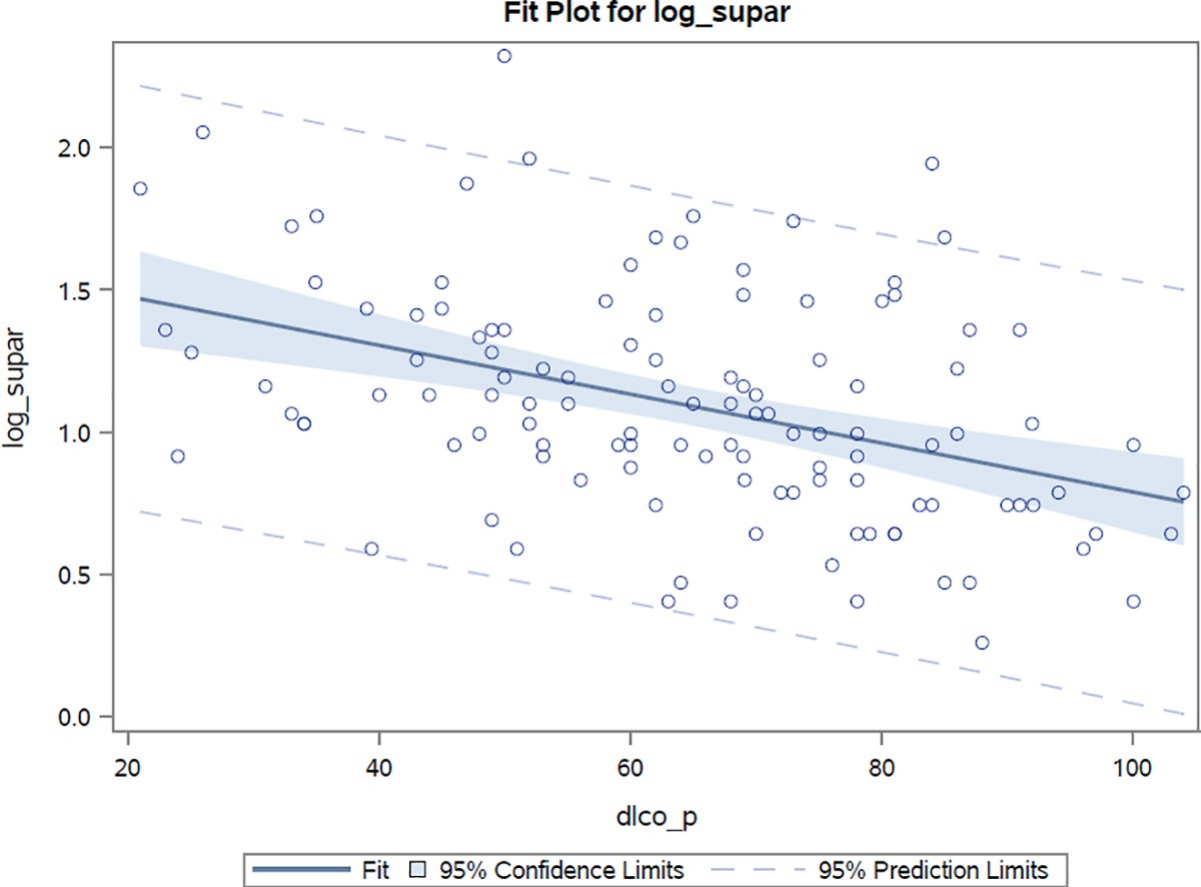
Correlation between log(suPAR) values and percentage of predicted diffusion capacity estimates. Correlation coefficient r = -0.41 *p*<0.0001.

**Fig 3:**
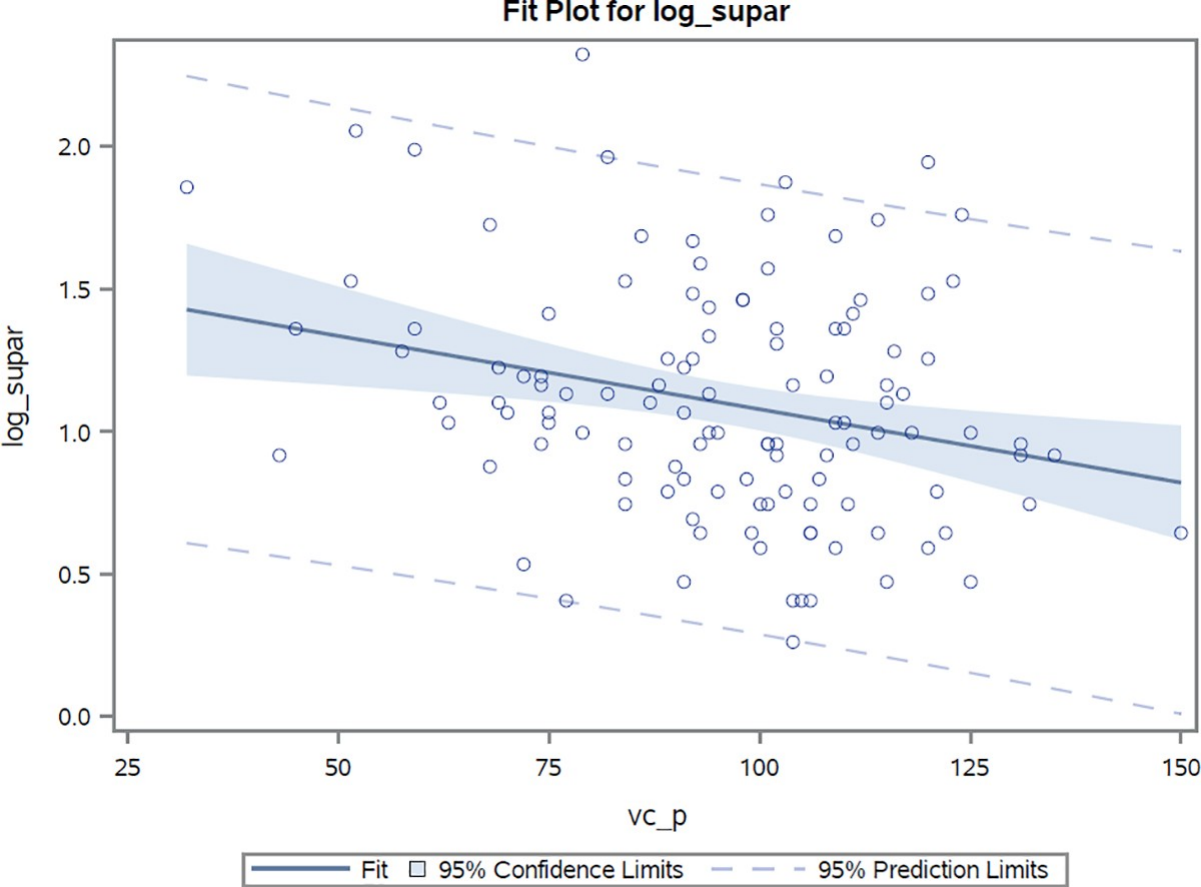
Correlation between log(suPAR) values and percentage of predicted total capacity estimates. Correlation coefficient r = -0.27 p<0.004.

Markers of pulmonary involvement (radiographically confirmed involvement, FVC, DLco) were associated with suPAR. Log(suPAR) values were found to demonstrate a high inverse correlation with both carbon monoxide diffusion capacity (r = -0.41, r^2^ = 0.17, *p* <0.0001) and forced vital capacity (r = -0.26, r^2^ = 0.07, *p* = 0.004) values, **Figs 2 and 3**. As compared to suPAR, log(hsCRP) was observed to be more closely related to forced vital capacity (r = -0.40, r^2^ = 0.16, *p* <0.0001) than to diffusion capacity (r = -0.26, r^2^ = 0.07, *p* = 0.006) estimates. After adjustments for age, sex and presence of pulmonary hypertension, the log(suPAR) level was found to be associated with percentage values of predicted diffusion capacity and forced vital capacity [β: -16.8% (*p* <0.0001) and -12.7 (*p* = 0.0005) per unit increase in log(suPAR), respectively]. After performing similar adjustments, hsCRP values were also observed to be associated with percentage values of both predicted diffusion capacity (beta estimate: -3.8%, *p* = 0.01) and forced vital capacity (beta estimate: -6.4%, *p* <0.0001) for every unit increase in the log(hsCRP) estimate.

## Discussion

We investigated the association between suPAR concentrations and organ-level involvement in patients with SSc. We found a strong correlation between suPAR levels and surrogate markers of interstitial lung disease in SSc patients. Conversely, we did not observe a correlation of this biomarker with the extent of dermal fibrosis (defined as per the modified Rodnan skin score), serological markers (anti-Scl-, centromere-, and RNA-polymerase-III-antibodies), or vascular manifestations (pulmonary hypertension, telangiectasias, systolic blood pressure, past or present cutaneous ulcers). The detailed relationship between fibrotic and vascular dysfunction in SSc remains unclear and the pathophysiological overlap is incompletely understood. Because of the heterogeneity and spectrum of organ involvement in SSc, there is currently no single biomarker that can serve as an indicator of disease activity or severity for all phenotypes of the condition. Earlier studies have suggested suPAR as a valuable marker of organ damage or disease activity in various conditions including systemic lupus erythematosus [28–30], rheumatoid arthritis [31, 32], liver [33], and kidney [15] disease. It has been shown to correlate well with mortality and prognosis in a wide range of patient populations [34–37]. The few studies that have been conducted show that levels of suPAR have also been found to be elevated in SSc patients as compared to those of healthy controls [19, 38]. Abnormalities in the uPA or uPAR systems in endothelial cells have been implicated in vascular complications related to SSc [39–41], and suPAR has been described as a marker of cardiovascular risk and mortality [26, 42], We did not find a difference in log(suPAR) levels in SSc patients with and without vascular manifestations (pulmonary hypertension, telangiectasias, past or present digital ulcers). Although we did not have a healthy comparison cohort, our results are in contrast to those of a previous study [19], wherein suPAR levels were found to be higher in SSc patients with pulmonary arterial hypertension (determined using right heart catheterization) and digital ulcers than in patients without these manifestations. Pulmonary hypertension in our cohort was determined on echocardiography, limiting diagnostic accuracy as compared to that achieved with right heart catheterization [43], which can be used to differentiate pulmonary arterial hypertension from other inciting conditions such as pulmonary obstructive disorders, interstitial lung disease and chronic thromboembolism.

Experimental studies suggest that uPAR may be causally involved in dermal, renal and lung fibrosis in mice (as evidenced by gene deficiency or inactivation studies) [13, 44, 45]. A recent human study, however, found no significant difference in suPAR concentrations among SSc patients with and without fibrotic gastrointestinal involvement, or according to their extent of dermal involvement (evaluated using the modified Rodnan skin score) [19]. However, the same study found significantly higher suPAR levels in patients with interstitial lung disease (determined using high-resolution CT scans), as compared to that in individuals without, suggesting that this biomarker may be more closely related to the pulmonary manifestations of SSc than with the involvement of other organs; high suPAR levels, in particular, correlated with severe reductions in the DLco by <40% [median suPAR value: 6.14; interquartile range (IQR): 4.68–6.45 ng/ml] and with moderate reductions in FVC (50% <FVC <69%) (median suPAR value: 5.18; IQR: 3.91–6.42 ng/ml). Our study corroborates these earlier findings, underscoring the implication that suPAR levels appear to have a close correlation with the severity of interstitial lung involvement. Although further studies are warranted to investigate if estimation of suPAR concentration can be used as a screening tool to detect pulmonary involvement in patients, our exploratory analyses indicates that a suPAR concentration ≤2.5 ng/mL could be used to rule out clinically relevant pulmonary involvement in SSc.

It is important to establish biomarker validity and utility [46]. Estimation of suPAR has been demonstrated to have analytical validity (accuracy, reproducibility and reliability) in a number of studies [15, 37]. Our Western European cohort of SSc patients was clinically well-characterized and sampled under identical and controlled conditions, strengthening the reliability of our data. Nevertheless, the present study has certain limitations. First, our study findings can only lead us to hypothesize as to the clinical validity of using suPAR estimations in stratifying the degree of interstitial lung involvement in SSc. Although suPAR is a relatively stable biomarker and SSc a chronic disease, pulmonary function testing, echocardiography and high-resolution computed tomography did not take place at the same time, which represents a limitation on the exact correlation with suPAR. Therefore, whether or not suPAR estimation has clinical utility (implying that biomarker usage can result in better or similar outcomes at lower cost) as a diagnostic and prognostic biomarker in SSc cannot be extrapolated from the findings of our study. Second, the number of patients in our study cohort was low, which decreased the power of discrimination between degrees of disease severity. Finally, we did not have a healthy control cohort for comparison, which would have helped us demonstrate the screening and diagnostic capability of the biomarker for detection of SSc-related organ manifestations.

In conclusion, our study shows that the plasma suPAR-concentrations may reflect interstitial lung disease severity and extent of organ damage in SSc patients. Our findings also indicate the possibility of utilization of suPAR levels to detect the extent of lung involvement in SSc, which in turn can correlate with future prognosis of the affected patient. Our findings may facilitate the development of novel biomarkers for non-invasive disease staging, though large-scale, prospective studies are warranted to investigate the possibility of utilizing suPAR as a marker for screening, monitoring and predicting prognosis in SSc patients.

## References

[1] Butt SA, Jeppesen JL, Torp-Pedersen C, Sam F, Gislason GH, Jacobsen S, et al Cardiovascular Manifestations of Systemic Sclerosis: A Danish Nationwide Cohort Study. J Am Heart Assoc. 2019;8(17):e013405 10.1161/JAHA.119.013405 31446827 PMC6755829

[2] Butt SA, Jeppesen JL, Fuchs C, Mogensen M, Engelhart M, Torp-Pedersen C, et al Trends in incidence, mortality, and causes of death associated with systemic sclerosis in Denmark between 1995 and 2015: a nationwide cohort study. BMC Rheumatol. 2018;2:36 10.1186/s41927-018-0043-6 30886986 PMC6390621

[3] Manetti M. Emerging biomarkers in systemic sclerosis. Curr Opin Rheumatol. 2016;28(6):606–12. 10.1097/BOR.0000000000000324 27380110

[4] Jog NR, James JA. Biomarkers in connective tissue diseases. J Allergy Clin Immunol. 2017;140(6):1473–83. 10.1016/j.jaci.2017.10.003 29221579 PMC5819750

[5] Kumanovics G, Gorbe E, Minier T, Simon D, Berki T, Czirjak L. Follow-up of serum KL-6 lung fibrosis biomarker levels in 173 patients with systemic sclerosis. Clin Exp Rheumatol. 2014;32(6 Suppl 86):S-138–44. 24773853

[6] Kuzumi A, Yoshizaki A, Toyama S, Fukasawa T, Ebata S, Nakamura K, et al Serum interleukin-34 levels in patients with systemic sclerosis: Clinical association with interstitial lung disease. J Dermatol. 2018;45(10):1216–20. 10.1111/1346-8138.14538 30004593

[7] Affandi AJ, Radstake TR, Marut W. Update on biomarkers in systemic sclerosis: tools for diagnosis and treatment. Semin Immunopathol. 2015;37(5):475–87. 10.1007/s00281-015-0506-4 26168983 PMC4554742

[8] Muangchan C, Harding S, Khimdas S, Bonner A, Canadian Scleroderma Research g, Baron M, et al Association of C-reactive protein with high disease activity in systemic sclerosis: results from the Canadian Scleroderma Research Group. Arthritis Care Res (Hoboken). 2012;64(9):1405–14. 10.1002/acr.21716 22556030

[9] Liu X, Mayes MD, Pedroza C, Draeger HT, Gonzalez EB, Harper BE, et al Does C-reactive protein predict the long-term progression of interstitial lung disease and survival in patients with early systemic sclerosis? Arthritis Care Res (Hoboken). 2013;65(8):1375–80. 10.1002/acr.21968 23401350 PMC3816494

[10] Lis-Swiety A, Widuchowska M, Brzezinska-Wcislo L, Kucharz E. High acute phase protein levels correlate with pulmonary and skin involvement in patients with diffuse systemic sclerosis. J Int Med Res. 2018;46(4):1634–9. 10.1177/0300060518760955 29512396 PMC6091829

[11] Muangchant C, Pope JE. The significance of interleukin-6 and C-reactive protein in systemic sclerosis: a systematic literature review. Clin Exp Rheumatol. 2013;31(2 Suppl 76):122–34. 23910616

[12] Ross L, Stevens W, Rabusa C, Wilson M, Ferdowsi N, Walker J, et al The role of inflammatory markers in assessment of disease activity in systemic sclerosis. Clin Exp Rheumatol. 2018;36 Suppl 113(4):126–34. 30277869

[13] Manetti M, Rosa I, Milia AF, Guiducci S, Carmeliet P, Ibba-Manneschi L, et al Inactivation of urokinase-type plasminogen activator receptor (uPAR) gene induces dermal and pulmonary fibrosis and peripheral microvasculopathy in mice: a new model of experimental scleroderma? Ann Rheum Dis. 2014;73(9):1700–9. 10.1136/annrheumdis-2013-203706 23852693

[14] Bernstein AM, Twining SS, Warejcka DJ, Tall E, Masur SK. Urokinase receptor cleavage: a crucial step in fibroblast-to-myofibroblast differentiation. Mol Biol Cell. 2007;18(7):2716–27. 10.1091/mbc.e06-10-0912 17507651 PMC1924808

[15] Hamie L, Daoud G, Nemer G, Nammour T, El Chediak A, Uthman IW, et al SuPAR, an emerging biomarker in kidney and inflammatory diseases. Postgrad Med J. 2018;94(1115):517–24. 10.1136/postgradmedj-2018-135839 30177549

[16] Andersen O, Eugen-Olsen J, Kofoed K, Iversen J, Haugaard SB. Soluble urokinase plasminogen activator receptor is a marker of dysmetabolism in HIV-infected patients receiving highly active antiretroviral therapy. J Med Virol. 2008;80(2):209–16. 10.1002/jmv.21114 18098145

[17] Kofoed K, Schneider UV, Scheel T, Andersen O, Eugen-Olsen J. Development and validation of a multiplex add-on assay for sepsis biomarkers using xMAP technology. Clin Chem. 2006;52(7):1284–93. 10.1373/clinchem.2006.067595 16690735

[18] Thuno M, Macho B, Eugen-Olsen J. suPAR: the molecular crystal ball. Dis Markers. 2009;27(3):157–72. 10.3233/DMA-2009-0657 19893210 PMC3835059

[19] Legany N, Toldi G, Distler JH, Beyer C, Szalay B, Kovacs L, et al Increased plasma soluble urokinase plasminogen activator receptor levels in systemic sclerosis: possible association with microvascular abnormalities and extent of fibrosis. Clin Chem Lab Med. 2015;53(11):1799–805. 10.1515/cclm-2015-0079 25894644

[20] Preliminary criteria for the classification of systemic sclerosis (scleroderma). Subcommittee for scleroderma criteria of the American Rheumatism Association Diagnostic and Therapeutic Criteria Committee. Arthritis Rheum. 1980;23(5):581–90. 10.1002/art.1780230510 7378088

[21] Iversen LV, Ostergaard O, Ullman S, Nielsen CT, Halberg P, Karlsmark T, et al Circulating microparticles and plasma levels of soluble E- and P-selectins in patients with systemic sclerosis. Scand J Rheumatol. 2013;42(6):473–82. 10.3109/03009742.2013.796403 24016306

[22] Iversen LV, Ullman S, Ostergaard O, Nielsen CT, Halberg P, Karlsmark T, et al Cross-sectional study of soluble selectins, fractions of circulating microparticles and their relationship to lung and skin involvement in systemic sclerosis. BMC Musculoskelet Disord. 2015;16:191 10.1186/s12891-015-0653-8 26265409 PMC4534013

[23] Hanitsch LG, Burmester GR, Witt C, Hunzelmann N, Genth E, Krieg T, et al Skin sclerosis is only of limited value to identify SSc patients with severe manifestations—an analysis of a distinct patient subgroup of the German Systemic Sclerosis Network (DNSS) Register. Rheumatology (Oxford). 2009;48(1):70–3. 10.1093/rheumatology/ken408 19056798

[24] Clements P, Lachenbruch P, Siebold J, White B, Weiner S, Martin R, et al Inter and intraobserver variability of total skin thickness score (modified Rodnan TSS) in systemic sclerosis. J Rheumatol. 1995;22(7):1281–5. 7562759

[25] LeRoy EC, Black C, Fleischmajer R, Jablonska S, Krieg T, Medsger TA Jr., et al Scleroderma (systemic sclerosis): classification, subsets and pathogenesis. J Rheumatol. 1988;15(2):202–5. 3361530

[26] Eugen-Olsen J, Andersen O, Linneberg A, Ladelund S, Hansen TW, Langkilde A, et al Circulating soluble urokinase plasminogen activator receptor predicts cancer, cardiovascular disease, diabetes and mortality in the general population. J Intern Med. 2010;268(3):296–308. 10.1111/j.1365-2796.2010.02252.x 20561148

[27] Lyngbaek S, Sehestedt T, Marott JL, Hansen TW, Olsen MH, Andersen O, et al CRP and suPAR are differently related to anthropometry and subclinical organ damage. Int J Cardiol. 2013;167(3):781–5. 10.1016/j.ijcard.2012.03.040 22459389

[28] Toldi G, Szalay B, Beko G, Bocskai M, Deak M, Kovacs L, et al Plasma soluble urokinase plasminogen activator receptor (suPAR) levels in systemic lupus erythematosus. Biomarkers. 2012;17(8):758–63. 10.3109/1354750X.2012.728623 23033975

[29] Enocsson H, Wettero J, Skogh T, Sjowall C. Soluble urokinase plasminogen activator receptor levels reflect organ damage in systemic lupus erythematosus. Transl Res. 2013;162(5):287–96. 10.1016/j.trsl.2013.07.003 23916811

[30] Wen S, He F, Zhu X, Yuan S, Liu H, Sun L. IFN-gamma, CXCL16, uPAR: potential biomarkers for systemic lupus erythematosus. Clin Exp Rheumatol. 2018;36(1):36–43. 28628472

[31] Toldi G, Beko G, Kadar G, Macsai E, Kovacs L, Vasarhelyi B, et al Soluble urokinase plasminogen activator receptor (suPAR) in the assessment of inflammatory activity of rheumatoid arthritis patients in remission. Clin Chem Lab Med. 2013;51(2):327–32. 10.1515/cclm-2012-0221 22718576

[32] Slot O, Brunner N, Locht H, Oxholm P, Stephens RW. Soluble urokinase plasminogen activator receptor in plasma of patients with inflammatory rheumatic disorders: increased concentrations in rheumatoid arthritis. Ann Rheum Dis. 1999;58(8):488–92. 10.1136/ard.58.8.488 10419867 PMC1752924

[33] Zimmermann HW, Koch A, Seidler S, Trautwein C, Tacke F. Circulating soluble urokinase plasminogen activator is elevated in patients with chronic liver disease, discriminates stage and aetiology of cirrhosis and predicts prognosis. Liver Int. 2012;32(3):500–9. 10.1111/j.1478-3231.2011.02665.x 22098627

[34] Godtfredsen NS, Jorgensen DV, Marsaa K, Ulrik CS, Andersen O, Eugen-Olsen J, et al Soluble urokinase plasminogen activator receptor predicts mortality in exacerbated COPD. Respir Res. 2018;19(1):97 10.1186/s12931-018-0803-2 29783959 PMC5963104

[35] Loosen SH, Tacke F, Binnebosel M, Leyh C, Vucur M, Heitkamp F, et al Serum levels of soluble urokinase plasminogen activator receptor (suPAR) predict outcome after resection of colorectal liver metastases. Oncotarget. 2018;9(43):27027–38. 10.18632/oncotarget.25471 29930748 PMC6007468

[36] Meyer J, Alstrup M, Rasmussen LJH, Schultz M, Ladelund S, Haupt TH, et al suPAR is associated with risk of future acute surgery and post-operative mortality in acutely admitted medical patients. Scand J Trauma Resusc Emerg Med. 2018;26(1):11 10.1186/s13049-018-0478-1 29391054 PMC5796401

[37] Eugen-Olsen J, Giamarellos-Bourboulis EJ. suPAR: The unspecific marker for disease presence, severity and prognosis. Int J Antimicrob Agents. 2015;46 Suppl 1:S33–4. 10.1016/j.ijantimicag.2015.10.011 26603640

[38] Bandinelli F, Bartoli F, Perfetto E, Del Rosso A, Moggi-Pignone A, Guiducci S, et al The fibrinolytic system components are increased in systemic sclerosis and modulated by Alprostadil (alpha1 ciclodestryn). Clin Exp Rheumatol. 2005;23(5):671–7. 16173244

[39] D’Alessio S, Fibbi G, Cinelli M, Guiducci S, Del Rosso A, Margheri F, et al Matrix metalloproteinase 12-dependent cleavage of urokinase receptor in systemic sclerosis microvascular endothelial cells results in impaired angiogenesis. Arthritis Rheum. 2004;50(10):3275–85. 10.1002/art.20562 15476218

[40] Margheri F, Manetti M, Serrati S, Nosi D, Pucci M, Matucci-Cerinic M, et al Domain 1 of the urokinase-type plasminogen activator receptor is required for its morphologic and functional, beta2 integrin-mediated connection with actin cytoskeleton in human microvascular endothelial cells: failure of association in systemic sclerosis endothelial cells. Arthritis Rheum. 2006;54(12):3926–38. 10.1002/art.22263 17133606

[41] Manetti M, Allanore Y, Revillod L, Fatini C, Guiducci S, Cuomo G, et al A genetic variation located in the promoter region of the UPAR (CD87) gene is associated with the vascular complications of systemic sclerosis. Arthritis Rheum. 2011;63(1):247–56. 10.1002/art.30101 20967855

[42] Lyngbaek S, Marott JL, Sehestedt T, Hansen TW, Olsen MH, Andersen O, et al Cardiovascular risk prediction in the general population with use of suPAR, CRP, and Framingham Risk Score. Int J Cardiol. 2013;167(6):2904–11. 10.1016/j.ijcard.2012.07.018 22909410

[43] Augustine DX, Coates-Bradshaw LD, Willis J, Harkness A, Ring L, Grapsa J, et al Echocardiographic assessment of pulmonary hypertension: a guideline protocol from the British Society of Echocardiography. Echo Res Pract. 2018;5(3):G11–G24. 10.1530/ERP-17-0071 30012832 PMC6055509

[44] Kanno Y, Kaneiwa A, Minamida M, Kanno M, Tomogane K, Takeuchi K, et al The absence of uPAR is associated with the progression of dermal fibrosis. J Invest Dermatol. 2008;128(12):2792–7. 10.1038/jid.2008.157 18548111

[45] Zhang G, Kim H, Cai X, Lopez-Guisa JM, Alpers CE, Liu Y, et al Urokinase receptor deficiency accelerates renal fibrosis in obstructive nephropathy. J Am Soc Nephrol. 2003;14(5):1254–71. 10.1097/01.asn.0000064292.37793.fb 12707394

[46] Hayes DF. Biomarker validation and testing. Mol Oncol. 2015;9(5):960–6. 10.1016/j.molonc.2014.10.004 25458054 PMC5528748

